# Ungulate malaria parasites

**DOI:** 10.1038/srep23230

**Published:** 2016-03-21

**Authors:** Thomas J. Templeton, Masahito Asada, Montakan Jiratanh, Sohta A. Ishikawa, Sonthaya Tiawsirisup, Thillaiampalam Sivakumar, Boniface Namangala, Mika Takeda, Kingdao Mohkaew, Supawan Ngamjituea, Noboru Inoue, Chihiro Sugimoto, Yuji Inagaki, Yasuhiko Suzuki, Naoaki Yokoyama, Morakot Kaewthamasorn, Osamu Kaneko

**Affiliations:** 1 Department of Protozoology, Institute of Tropical Medicine (NEKKEN), Nagasaki University, 1-12-4 Sakamoto, Nagasaki 852-8523, Japan; 2 Department of Microbiology and Immunology, Weill Cornell Medical School, New York, New York, 10021, USA; 3 Parasitology Section, National Institute of Animal Health, Department of Livestock Development, Bangkhen, Bangkok 10900, Thailand; 4 Faculty of Life and Environmental Sciences, University of Tsukuba, Tsukuba, Ibaraki 305-8577, Japan; 5 Center for Computational Sciences, University of Tsukuba, Tsukuba, Ibaraki 305-8577, Japan; 6 Animal Vector-Borne Diseases Research Group, The Veterinary Parasitology Unit, Department of Pathology, Faculty of Veterinary Science, Chulalongkorn University, Bangkok 10330, Thailand; 7 National Research Center for Protozoan Diseases, Obihiro University of Agriculture and Veterinary Medicine, Inada-cho, Obihiro, Hokkaido 080-8555, Japan; 8 Department of Paraclinical Studies, School of Veterinary Medicine, University of Zambia, P.O. Box 32379 Lusaka, Zambia; 9 Research Center for Zoonosis Control, Hokkaido University, Sapporo, Hokkaido 060-0818, Japan; 10 Graduate School of Life and Environmental Sciences, University of Tsukuba, Tsukuba, Ibaraki 305-8577, Japan; 11 The Veterinary Parasitology Unit, Department of Pathology, Faculty of Veterinary Science, Chulalongkorn University, Bangkok 10330, Thailand

## Abstract

Haemosporida parasites of even-toed ungulates are diverse and globally distributed, but since their discovery in 1913 their characterization has relied exclusively on microscopy-based descriptions. In order to bring molecular approaches to bear on the identity and evolutionary relationships of ungulate malaria parasites, we conducted *Plasmodium cytb*-specific nested PCR surveys using blood from water buffalo in Vietnam and Thailand, and goats in Zambia. We found that *Plasmodium* is readily detectable from water buffalo in these countries, indicating that buffalo *Plasmodium* is distributed in a wider region than India, which is the only area in which buffalo *Plasmodium* has been reported. Two types (I and II) of *Plasmodium* sequences were identified from water buffalo and a third type (III) was isolated from goat. Morphology of the parasite was confirmed in Giemsa-reagent stained blood smears for the Type I sample. Complete mitochondrial DNA sequences were isolated and used to infer a phylogeny in which ungulate malaria parasites form a monophyletic clade within the Haemosporida, and branch prior to the clade containing bird, lizard and other mammalian *Plasmodium*. Thus it is likely that host switching of *Plasmodium* from birds to mammals occurred multiple times, with a switch to ungulates independently from other mammalian *Plasmodium.*

Malaria parasites of even-toed ungulates were first observed in 1913 by Sir David Bruce during a survey of blood parasites of duiker antelope in Malawi^1^. The parasite, which he named *Plasmodium cephalophi*, was not revisited and confirmed until a 1965 survey in the same region^2^,^3^. Several years after the first identification of *P. cephalophi*, a malaria parasite of domestic water buffalo was identified in India and named *Plasmodium bubalis*^4^. The literature on *P. bubalis* is solely comprised of veterinary cases of likely immunocompromised water buffalo, accompanied by microscopic descriptions of the parasite^5^,^6^,^7^,^8^,^9^,^10^. Other Haemosporida parasites of ungulates have been described, such as *Plasmodium traguli* and *Hepatocystis fieldi* in the Asian mouse deer^11^,^12^, a *Hepatocystis* parasite in the hippopotamus^13^, *Plasmodium limnotragi* in the African marshbuck^14^, and *Plasmodium caprae* in African goats^15^. Most recently, although over three decades ago, a malaria parasite was identified in blood smears of a white-tailed deer in North America, and named *Plasmodium odocoilei*^16^. Although Haemosporida parasites of even-toed ungulates are diverse and include both New and Old World species, their characterization has relied exclusively on microscopy-based descriptions and no molecular or cellular studies have been conducted. Herein we use a *cytb*-specific nested PCR assay to survey the presence of *Plasmodium* parasites in water buffalo in Asia and goats in Zambia, as well as isolation of whole mtDNA and *clpc* sequences in order to infer the evolutionary relationships of ungulate malaria parasites within the Haemosporida.

## Results and Discussion

**Figure 1:**
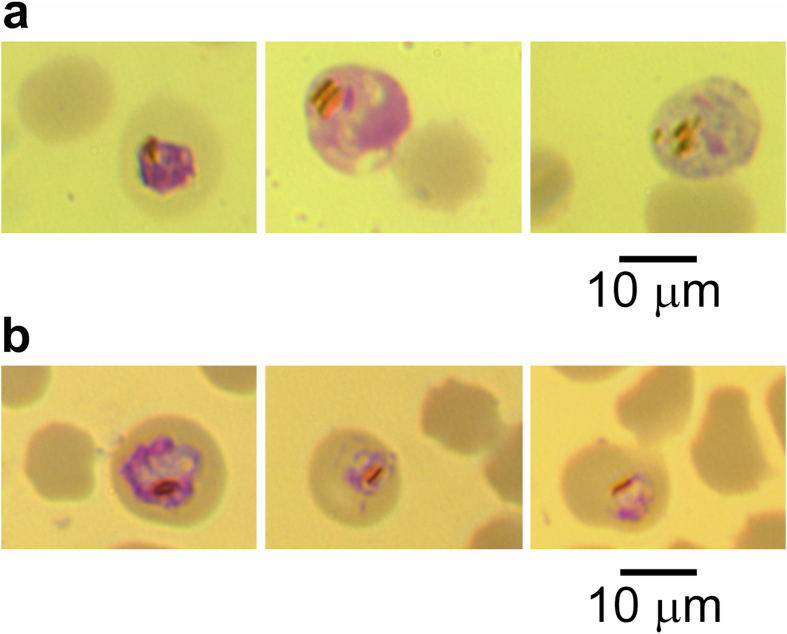
Bright field microscope images of blood smears stained with Giemsa reagent. Predicted *P. bubalis* parasites observed in (**a**), blood sample from a sick water buffalo in the Chachoengsao Province of Thailand in 2008; and (**b**), blood sample from a water buffalo in the Mukdahan Province of Thailand in 2015. Type-specific PCR of DNA isolated from the blood of this buffalo indicated that the parasite is Type I.

In 2008 we observed malaria parasites in Giemsa reagent-stained slides of blood smears prepared during examination of a sick domestic water buffalo (*Bubalus bubalis*) in the Chachoengsao Province of Thailand. Infected erythrocytes, including male and female gametocytes, were readily seen by microscopy (Fig. 1a; additional images in Supplementary Fig. S1a), with a parasitemia of less than 0.05%. The presence of hemozoin crystals were diagnostic of malaria parasites, and were frequently rod-shaped, as described by Sheather (1919) for *Plasmodium bubalis* (Supplementary Fig. S1b). Infected erythrocytes appeared larger and spherical, as if swollen, and hyaline in appearance compared with normal erythrocytes. Schizonts were not observed, possibly due to sequestration in the microvasculature away from the general circulation. Because only a single *Plasmodium* species has been described, in domestic water buffalo in India, the parasite is herein provisionally named *P. bubalis*.

To characterize ungulate malaria parasites at a molecular level, the *Plasmodium* mitochondrial cytochrome b (*cytb*) gene was amplified by PCR from DNA purified from blood collected in the course of four surveys: i) 92 archived blood DNA samples sourced from water buffaloes in the Thua Thien Hue province of Vietnam in 2010 and 2013^17^,^18^,^19^; ii) 95 DNA samples isolated from dried blood spots on filter papers from water buffalo in the Amphoe Khamcha-I district of Thailand in 2014; iii) 144 DNA samples isolated from freshly drawn blood from a water buffalo survey in the Amphoe Mueang Mukdahan district of Thailand in 2015; and iv) 53 DNA samples purified from the blood of goats in Zambia^20^. PCR products were sequenced and two distinct *cytb* gene sequences were identified from water buffalo, which we provisionally refer to as *P. bubalis* Types I and II. Table 1 provides a compendium of the surveys, including the percent positivity and prevalence of Types I and II as assayed by nested PCR. Type-specific PCR assays, as well as *cytb* nucleotide sequencing trace analyses, indicated that some buffalo were co-infected with Types I and II. Survey of 53 goats in Zambia identified a single positive sample, which yielded a *cytb* type distinct from Types I and II and is herein referred to as Type III. One report from the year 1923 describes a parasite, *Laverania caprae*^15^, renamed *Plasmodium caprae*^21^, in African goats; and thereby we provisionally name the Type III parasite *P. caprae*. Giemsa reagent stains of blood smears were not obtained for this survey of Zambian goats, and it is therefore not possible to provide a morphological description.

The third survey described above, the isolation of DNA from freshly collected buffalo blood in Thailand, included matched preparation of blood smears on glass slides. Microscope survey of Giemsa reagent-stained blood smears from 65 buffalo, determined to be positive for *P. bubalis* by diagnostic PCR assay, did not yield observation of infected erythrocytes. This concurs with reports that the parasite is occult in the absence of an immunocompromised state, such as proposed regarding the observation of *P. bubalis* in water buffalo which were used for the derivation of immune sera for rinderpest^5^,^6^,^7^. Therefore, as a proxy for visual determination of parasitemia, we designed a quantitative PCR assay (qPCR) of *P. bubalis* mtDNA copy number, in order to identify buffalo which might harbor elevated parasite burdens. These assays identified 5 DNA samples, all from Type I infections, having qPCR signals significantly higher than the other *Plasmodium* mtDNA-positive samples. The Giemsa reagent-stained blood smear slide corresponding to the sample with the highest signal yielded infected parasites in a microscope survey (Fig. 1b; additional images in Supplementary Fig. S1c), although with a parasitemia of less than 0.003%. Bar-shaped hemozoin, characteristic of *P. bubalis* (Supplementary Fig. S1b), were seen in many parasites.

**Figure 2:**
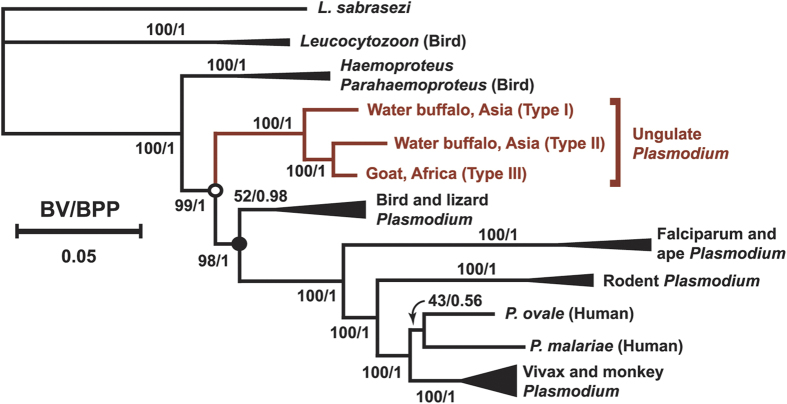
Phylogenetic relationships of ungulate malaria parasites within the Haemosporida. Ungulate malaria parasites group together and branch before diversification of malaria parasites that infect bird, lizard, rodent, monkey, apes and human. The tree was inferred by the maximum likelihood (ML) method based on the GTR + I + G model, by using 36 whole mitochondria genome sequences as a concatenated nucleotide sequence data. Bootstrap values (BV) by ML with 100 replicates and Bayesian posterior probability (BPP) by BI are indicated for each internal branch^34^,^35^. Collapsed clades are indicated, such as “Vivax and monkey *Plasmodium*” clade, and their compositions are described in the Methods section. The node at the split of the ungulate clade is indicated by an open circle and the node at base of the bird, lizard, and main mammalian clade is indicated by a closed circle. The length for the substitutions/site (0.05) is indicated.

**Figure 3:**
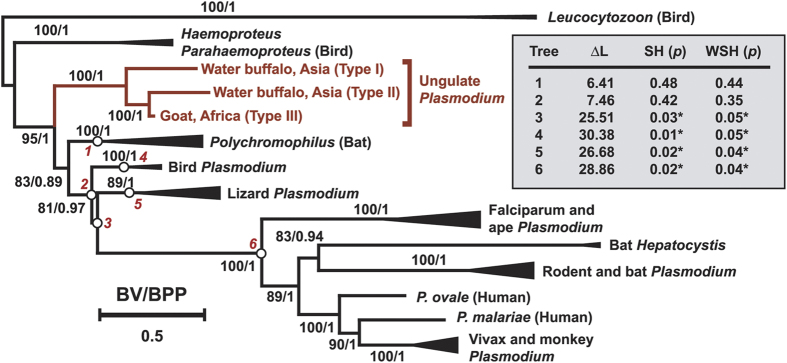
Phylogenetic relationships of ungulate *Plasmodium* within the Haemosporida using concatenated *coxI*, *cytb*, and *clpc* nucleotide sequences (codon model). Model analysis using ModelOMatic^36^ indicated that a codon model (AIC score 46566.5) is superior to the nucleotide and amino acid model (AIC score 50700.8 and 65635.5, respectively). Thus we analyzed the phylogenetic relationship using a codon model (GY + I + G + F)^37^. The maximum likelihood (ML) tree was inferred using concatenated *coxI*, *cytb*, and *clpc* gene sequences from 52 *Plasmodium* and related parasites (Supplementary Table S1). Gaps existing in equal to or greater than 50% of the sequences were excluded from the analysis. 0.5 indicates substitutions/site. Bootstrap values (BV) by ML with 1,000 replicates and Bayesian posterior probability (BPP) are indicated for each internal branch. The “fast bootstrap” method implemented in IQ-TREE was applied for the ML bootstrap analysis based on the codon model. The compositions of the collapsed clades are indicated in Supplementary Table S1. The BPP value between the ungulate *Plasmodium* clade and the *Polychromophilus* clade did not reach a value of 1.00. ML tree was examined (shaded inset) by regrafting the ungulate *Plasmodium* clade to the 6 nodes shown on the original ML tree, indicated by circles labeled with italicized red numbers. Values are shown for ΔL, log-likelihood difference to most likely ML tree in the set; SH, Shimodaira-Hasegawa test^38^; WSH, weighted SH test; *p*, p-value. Tests were performed with 10,000 resamplings using the RELL method. All tests rejected the hypotheses that ungulate *Plasmodium* and bird, lizard, or other mammalian *Plasmodium* are monophyletic (asterisks, *p* < 0.05). However, all tests failed to clarify the relationship between the ungulate *Plasmodium* and *Polychromophilus* clades, and thus this relationship warrants further investigation.

We were interested in determining the placement of ungulate malaria parasites within the Haemosporida phylogenetic tree and to facilitate these analyses we isolated the complete mtDNA nucleotide sequence (roughly 6 kbp) via PCR amplification from the DNA isolated from two infected Thailand water buffalo each for Types I and II, and the single Type III isolated from the Zambian goat. Within each type the mtDNA nucleotide sequence was 100% identical for the two isolated examples, with no polymorphisms, suggesting that composite mtDNA were not assembled. Polymorphisms were also not observed within type for *cytb* gene sequences obtained from 20 Type I and 39 Type II samples from water buffalo. Alignments of mtDNA nucleotide sequence were obtained and phylogenetic trees of Haemosporida were inferred by the maximum likelihood (ML) and Bayesian inference (BI) methods (Fig. 2 and Supplementary Fig. S2). The three types group together with strong bootstrap support and Bayesian posterior probability (100 and 1.00, respectively). Within this clade *P. bubalis* Type II is more closely related to the Type III *P. caprae* goat parasite than it is to *P. bubalis* Type I. The deep branching of the three types, which is greater than that of the rodent malaria parasites *P. yoelii* and *P. berghei* (Supplementary Fig. S3), supports the hypothesis that they are distinct species, lacking the ability to produce recombinant progeny. Isolation of complete genome sequence information will help to resolve the species designations, as well as description for *P. caprae* (Type III). The relationships among the different clades within *Plasmodium* are in agreement with recently published phylogenetic trees^22^,^23^,^24^,^25^, and within this structure the ungulate malaria parasite clade branches prior to the divergence of the bird, lizard and mammalian *Plasmodium* clades (supported by ML and BI with both concatenated and partitioned sequences). Because the bat haemosporidian parasite *Polychromophilus* is also proposed to branch out before the diversification of bird, lizard and mammalian malaria parasites, although not well resolved^25^,^26^,^27^, we analyzed the phylogenetic relationship between ungulate *Plasmodium* and *Polychromophilus.* The caseinolytic protease C gene (*clpc*) gene was obtained in order to increase the number of sequences from available parasite species including *Polychromophilus*. DNA fragments coding for *clpc* were amplified and nucleotide sequence determined from Type I and II water buffalo samples and a concatenated alignment of the open reading frames for *coxI, cytb* and *clpc* genes were used to infer phylogenetic trees of Haemosporida (Fig. 3). In this analysis *Polychromophilus* was also inferred to be branched out prior to the divergence of the bird, lizard and other mammalian *Plasmodium* clades, which is consistent with a recent report^25^; however, we were unable to obtain good support to describe the relationship of the ungulate *Plasmodium* clade with the *Polychromophilus* clade (Fig. 3 inset). Thus it remains unclear if *Polychromophilus* serves as further evidence for the paraphyletic nature of *Plasmodium*, such as currently proposed with respect to *Hepatocystis*^22^,^28^,^29^. When *cytb* nucleotide sequences for Types I, II or III are used as BLAST queries of GenBank then the best hit is a *cytb* sequence (sample S2138_CS8; GenBank accession JQ070884), isolated from a white-nosed monkey bushmeat survey in Cameroon^30^. Our phylogenetic analysis with 52 *Plasmodium* and related parasites places this sequence within the ungulate *Plasmodium* clade with an ML-bootstrap value of 99 and a Bayesian posterior probability of 1.00 (Supplementary Fig. S4); rather than the bird *Plasmodium* clade as reported^30^ and *Hepatocystis* as annotated in the GenBank entry. It should be deliberated if the sample S2138_CS8 parasite has a broad range of vertebrate hosts, including ungulates, or was misidentified in collection and was from an ungulate host, rather than a monkey.

This survey of *Plasmodium* in Asian water buffalo and Zambian goats via *cytb*-specific nested PCR and inference of phylogeny using whole mtDNA and *clpc* sequences represents the first molecular study on ungulate malaria parasites. The early branching of ungulate malaria parasites within Haemosporida raises the question of whether the vertebrate host was bird, lizard or mammal at the nodes corresponding to the ungulate *Plasmodium* branch and the main mammalian *Plasmodium* branch (indicated in Fig. 2 by open and closed circles, respectively). Because *Leucocytozoon* and *Haemoproteus/Parahaemoproteus* parasites infect birds and branch before the diversification of all *Plasmodium* groups and bat *Polychromophilus*, it is likely that host switching events from birds to mammals occurred multiple times. Pre-erythrocytic schizogony of ungulate malaria parasites occurs in hepatocytes^11^,^12^, versus other host cells such as reticuloendothelial or lung cells, as observed for avian Haemosporida and *Polychromophilus*. The vector for ungulate *Plasmodium* has been proposed to be an *Anopheles* mosquito^31^,^32^; however, other species should also be considered since, for example, bird and lizard *Plasmodium* are able to be transmitted by *Culex* and *Aedes*. *Polychromophilus*, in contrast, is transmitted by Hippobosoidea biting flies rather than mosquitoes.

Because ungulate *Plasmodium* can be obtained from domestic animals in a wide region spanning Asia and Africa, as found in this study, these parasites have potential to underpin a new malaria model. In this regard future studies on ungulate malaria parasites will benefit from either tissue culture adaptation of the parasite or the development of an erythrocyte “ungulate-ized” splenectomized SCID mouse model. Either method, as well as infections of splenectomized water buffalo or goats (for Type III/*P. caprae*) would enable purification of sufficient parasite material to allow whole genome sequencing, as well as to aid studies on the identities of insect vectors. Studies on *P. bubalis* would benefit from the ability to use cattle (*Bos taurus*) blood rather than water buffalo. However, we cannot find reports that cattle harbor malaria parasites, and we were unable to detect *Plasmodium* DNA in a survey of 595 DNA samples from cattle in Vietnam (123 samples) and Zambia (472 samples) using the *cytb* nested PCR assay. The derivation of whole genome sequence information for an ungulate malaria parasite would not be compromised by the presence of host gDNA contamination from nucleated erythrocytes, as has been encountered for bird malaria parasites. Once whole genome sequence is obtained it will be of particular interest to annotate genes that encode proteins predicted to be involved in virulence and pathogenesis, as a means to understanding the evolution of these proteins within the *Plasmodium* genus.

## Methods

### Sample collections

This study was initiated with an analysis of Giemsa reagent-stained slides of blood smears collected by the National Institute of Animal Health of Thailand during examination of a sick water buffalo in June, 2008. The buffalo was an indigenous and local breed from the Nong Mai Kaen locality in Plaeng Yao District, Chachoengsao Province near Bangkok of Thailand. Additional blood samples were collected in two surveys from two districts of Mukdahan Province near the northeast border of Thailand. Sample collections were part of an annual field trip of Chulalongkorn University Faculty of Veterinary Science in cooperation with the Department of Livestock Development of Thailand. In the first survey, during late March and early April of 2014, 95 blood samples were collected from indigenous water buffaloes, without discrimination of sex and age, and owned by small holder farmers in Khamcha-i District. Buffaloes were between 5 months to 7 years of age, with 85% over 1 year old, and 79% were female buffaloes. A total of 200 μl of each blood sample was drawn aseptically from the caudal vein at the base of the tail and spotted onto DNase-free Whatman 3 MM filter paper (Brentford, UK). Each sample was stored at room temperature in a separate sealed bag to avoid cross-contamination prior to DNA extraction. DNA extractions from the dried blood spots were performed using an EZ1 DNA Blood Kit (Qiagen^®^) according to the manufacturer’s instructions. In the second survey 144 samples were collected during late June in 2015 in the Mueang Mukdahan District. The buffalo ranged in age from 4 months to over 10 years; 98% were over 1 year old and 77% were female buffaloes. Blood samples were collected from 16 different villages or sites. Blood withdrawal was from the jugular vein of buffalo restrained within squeeze pens; using aseptic procedures to draw approximately 8 ml of blood into BD Vacutainer^®^ ACD solution A (BD Franklin Lakes, NJ, USA). Blood samples were then divided for making thin blood smears and freezing for DNA extraction. For DNA extraction 200 μl of blood was treated on ice with 800 μl of 0.15% saponin in 1X PBS for a few minutes and then centrifuged at 1600 × g for 2 minutes. Supernatants of reddish color were discarded and the pellet further washed with 1X PBS until the supernatant turned clear. The pellet was then subjected to DNA extraction using a QIAamp^®^ DNA Mini Kit (Qiagen^®^) following the manufacturer’s instructions, with a final elution volume of 50 μl. Both surveys were conducted with the consent of the buffalo owners and were approved by the Institutional Animal Care and Use Committee (no. 1531058) and carried out in accordance with the approved guidelines. Goat samples were archived DNA collected in 2010 from Chama village in northeastern Zambia as described^20^.

### Plasmodium cytb assays

The *Plasmodium cytb* gene was amplified by nested PCR using the *Plasmodium* “universal” primers DW2 (TAATGCCTAGACGTATTCCTGATTATCCAG) and DW4 (TGTTTGCTTGGGAGCTGTAATCATAATGTG) for the primary PCR reaction, and NCYBINF (TAAGAGAATTATGGAGTGGATGGTG) and NCYBINR (CTTGTGGTAATTGACATCCAATCC) for the nested PCR reaction as described^33^. The PCR reaction conditions were 35 cycles of denaturation and 62 °C for 3 minutes, with no hybridization step; and typically 40 fold dilution of the primary PCR product within the secondary reaction. DNA templates used in the PCR screen for *Plasmodium* were i) from archived samples from Vietnam water buffalo as described^17^,^18^,^19^; ii) isolated from blood spots on filter paper drawn from water buffalo in Thailand in 2014, described above; iii) isolated from fresh blood from water buffalo in Thailand in 2015, described above; and iv) 53 goat DNA archived samples from northeastern Zambia. Type-specific nested PCR primers were used to determine the prevalence of Type I and Type II single and mixed infections. For these assays the *P. bubalis* universal primers used for the primary PCR amplification were TypeUnivFor (CGTGCTAAAGGTTTAACAC) and TypeUnivRev (ATTGAGTTATCGCGTAAATG); and the primers used for the nested PCR amplification were Type1ForInt (CCAGTTTTAACAGGTGGTGTA) and Type1RevInt (GCATTTCCTAATATAACTCCA) specific for Type I, and Type2ForInt (CCTGTATTAACTGGTGGAGTT) and Type2RevInt (GCATTACCTAAAATGACTCCT) specific for Type II. Complete mtDNA sequences were amplified using a panel of forward and reverse PCR primers designed based upon *Plasmodium* conserved nucleotide sequences, with weighting by eye using bird malaria parasite *P. gallinaceum* and *P. relictum* sequences. For each *P. bubalis* Type, nucleotide sequences were obtained from the sequencing of both DNA strands; and from DNA isolated from two buffalo. The above panel of primers was also used for sequencing of both strands of the Type III DNA from the Zambian goat blood DNA sample. The mitochondrial DNA and *clpc* DNA nucleotide sequences determined from ungulate *Plasmodium* in this study were deposited in DDBJ/EMBL/GenBank with the accession numbers LC090213–LC090217.

### Quantitative *Plasmodium cytb* assays

SYBR Green quantitative real-time PCR (qPCR) assays of parasite mitochondrial DNA were performed using DNA template from 65 buffalo which were positive by the above described nested PCR specific for *cytb*. For the qPCR assay the above TypeUnivFor primer and TypeUnivRevii (TATAATACTGGATCACCAGC) primer were used to amplify a 179 bp target region of the parasite *cytb* gene. Each 20 μl reaction contained 1 μl of template DNA solution, 10 μl of Power SYBR Green PCR Master Mix (Applied Biosystems), and each primer at a final concentration of 300 nM. Quantitative PCR reactions were performed on a 7500 Real Time PCR System (Applied Biosystems) using temperature profiles based on the manufacturer’s instructions and analysis using 7500 System SDS software (Applied Biosystems). The specificity of the amplification was confirmed by insertion of PCR products into the pCR-Blunt II-TOPO plasmid followed by nucleotide sequencing of the insert. The resulting plasmid was used to construct standard curves of gene copy number for the quantitative assay. Each sample and standard controls were amplified in triplicate with accompanying negative controls, and the resulting cycle threshold values were averaged across the three wells. The qPCR results showed that the Muk2015_52 sample contained the highest copy number (8.2 × 10^4^ copies/μl) followed by Muk2015_53 (1.5 × 10^4^ copies/μl). Three other samples had greater than 1 × 10^3^ copies/μl. The Muk2015_52 sample was then found to be positive for *Plasmodium* parasites by a microscopic survey of the respective Giemsa reagent-stained blood smear. To further confirm the identity of this parasite a PCR assay was conducted, and shown to be negative, using primers diagnostic for *Babesia* and *Theileria*, with matched positive controls.

### Phylogenetic analysis

Accession numbers for mitochondrial nucleotide sequences used to assess the phylogenetic position of the parasite DNA obtained from water buffalo and goat are listed in Supplementary Table S1. Sequences were aligned using MUSCLE software with manual corrections. Unrooted trees were inferred by the maximum likelihood (ML) method with *Leucocytozoon* sequences as an out-group using IQ-TREE ver.1.3.10^34^. The ML tree inference, as well as the bootstrap analysis with 100 replicates, was performed based on the substitution model showing the smallest AIC score for the sequence data of interest (indicated in the legend for each tree), which was selected among all models implemented in IQ-TREE. Bayesian posterior probabilities were obtained using MrBayes ver. 3.2.5^35^, applying the same substitution model as that used in the corresponding ML analysis. Eight parallel Metropolis-coupled Markov chain Monte Carlo runs, each consisting of one cold and four heated chains with a chain temperature of 0.1, were run for 1,000,000 generations. Log-likelihood scores and trees with branch lengths were sampled every 1,000 generations. The first 250,000 generations were excluded as burn-in, and the remaining trees were summarized to obtain Bayesian posterior probabilities. In both the ML and BI analyses, the among-site rate variation was approximated with a discrete gamma distribution with four categories (+Γ option), and the proportion of invariant site was also estimated (+I option). DNA fragments coding for *clpc* were amplified and nucleotide sequenced determined from Type I and II water buffalo samples (2014 Mukdahan Province survey) by semi-nested PCR using forward primer CLPC.outF (GGTAAAACTGAATTAGCAAAAATA) and reverse primers CLPC.outR (CGAGCTCCATATAAAGGAT) and CLPC.inR (GAGCTCCATATAAAGGATTATAAG). The open reading frame from this sequence was concatenated with *coxI* and *cytb* open reading frames and subjected to the ML and BI analyses following the same procedure as described above. Sequences used for this analysis are summarized in the Supplementary Table S1.

The composition of the collapsed clades shown in Fig. 2 are as follows: “Vivax and monkey *Plasmodium*” clade includes *P. vivax*, *P. coatneyi*, *P. cynomolgi*, *P. fieldi*, *P. fragile*, *P. gonderi*, *P. hylobati*, *P. inui*, *P. knowlesi*, *P. simiovale* and *P. simium*; the “Rodent *Plasmodium*” clade includes *P. berghei*, *P. vinckei vinckei*, *P. yoelii* and *P. chabaudi*; the “Falciparum and ape *Plasmodium*” clade (also termed Laverania) includes *P. falciparum*, *P. reichenowi*, *P. billbrayi* and *P. billcollinsi*; the “Bird and lizard *Plasmodium*” clade includes *P. floridense*, *P. mexicanum*, *P. gallinaceum*, *P. relictum*, *P. juxtanucleare*, and *P. lutzi*; the “Haemoproteus/Parahaemoproteus” clade includes *Haemoproteus* spp. (jb1.JA27 and jb2.SEW5141) and *Parahaemoproteus vireonis*; and the “Leucocytozoon” clade includes *Leucocytozoon fringillarium*, *L. majoris* and *L. sabrasezi*.

## Additional Information

**How to cite this article**: Templeton, T. J. *et al.* Ungulate malaria parasites. *Sci. Rep.* **6**, 23230; doi: 10.1038/srep23230 (2016).

## Supplementary Material

Supplementary Information

## Figures and Tables

**Table 1:** *Plasmodium* prevalence and type as determined by *cytb* nested PCR assays.

Study	Host	Numberofsamples	Type Ipositive(%)	Type IIpositive(%)	MixedinfectionType Iand II	Type IIIpositive(%)	Totalpositive(%)
Thailand (2015)	Buffalo	144	8 (6%)	32 (22%)	25 (17%)	**–**	65 (45%)
Thailand (2014)	Buffalo	95	9 (9%)	5 (5%)	1 (1%)	**–**	15 (16%)
Vietnam (2013)	Buffalo	49	1 (2%)	2 (4%)	**–**	**–**	3 (6%)
Vietnam (2010)	Buffalo	43	2 (5%)	**–**	**–**	**–**	2 (5%)
Zambia (2010)	Goat	53	**–**	**–**	**–**	1 (2%)	1 (2%)
