# Supplementary material for: Ungulate malaria parasites
Source: Sci Rep. 2016 Mar 21;6:23230. doi: 10.1038/srep23230 (PMC4800408; doi:10.1038/srep23230)
Supplement: Supplementary Information [file srep23230-s1.pdf]

Ungulate malaria parasites

Thomas J. Templeton[1,2]*, Masahito Asada[1]*, Montakan Jiratanh[3], Sohta A. Ishikawa[4,5], Sonthaya Tiawsirisup[6], Thillaiampalam Sivakumar[7], Boniface Namangala[8], Mika Takeda[1], Kingdao Mohkaew[3], Supawan Ngamjituea[3], Noboru Inoue[7], Chihiro Sugimoto[9], Yuji Inagaki[5,10], Yasuhiko Suzuki[9], Naoaki Yokoyama[7], Morakot Kaewthamasorn[11] & Osamu Kaneko[1]

[1]Department of Protozoology, Institute of Tropical Medicine (NEKKEN), Nagasaki University, 1-12-4 Sakamoto, Nagasaki 852-8523, Japan
[2]Department of Microbiology and Immunology, Weill Cornell Medical School, New York, New York, 10021, USA
[3]Parasitology Section, National Institute of Animal Health, Department of Livestock Development, Bangkhen, Bangkok 10900, Thailand.
[4]Faculty of Life and Environmental Sciences, University of Tsukuba, Tsukuba, Ibaraki 305-8577, Japan
[5]Center for Computational Sciences, University of Tsukuba, Tsukuba, Ibaraki 305-8577, Japan.
[6]Animal Vector-Borne Diseases Research Group, The Veterinary Parasitology Unit, Department of Pathology, Faculty of Veterinary Science, Chulalongkorn University, Bangkok 10330, Thailand.
[7]National Research Center for Protozoan Diseases, Obihiro University of Agriculture and Veterinary Medicine, Inada-cho, Obihiro, Hokkaido 080-8555, Japan
[8]Department of Paraclinical Studies, School of Veterinary Medicine, University of Zambia, P.O. Box 32379 Lusaka, Zambia.
[9]Research Center for Zoonosis Control, Hokkaido University, Sapporo, Hokkaido 060-0818, Japan.
[10]Graduate School of Life and Environmental Sciences, University of Tsukuba, Tsukuba, Ibaraki 305-8577, Japan.
[11]The Veterinary Parasitology Unit, Department of Pathology, Faculty of Veterinary Science, Chulalongkorn University, Bangkok 10330, Thailand.

*These authors contributed equally to this work.
Correspondence and requests for materials should be addressed to M.K. (email: morakot.k@chula.ac.th) or O.K. (email: okaneko@nagasaki-u.ac.jp).

Supplementary Figures and Table.

References

1. Sheather, A. L. A malaria parasite in the blood of a buffalo. *J. Comp. Path. Ther.* **32,** 223-226 (1919).
2. Goldman, N. & Yang, Z. A codon-based model of nucleotide substitution for protein-coding DNA sequences. *Mol. Biol. Evol.* **11,** 725-736 (1994).

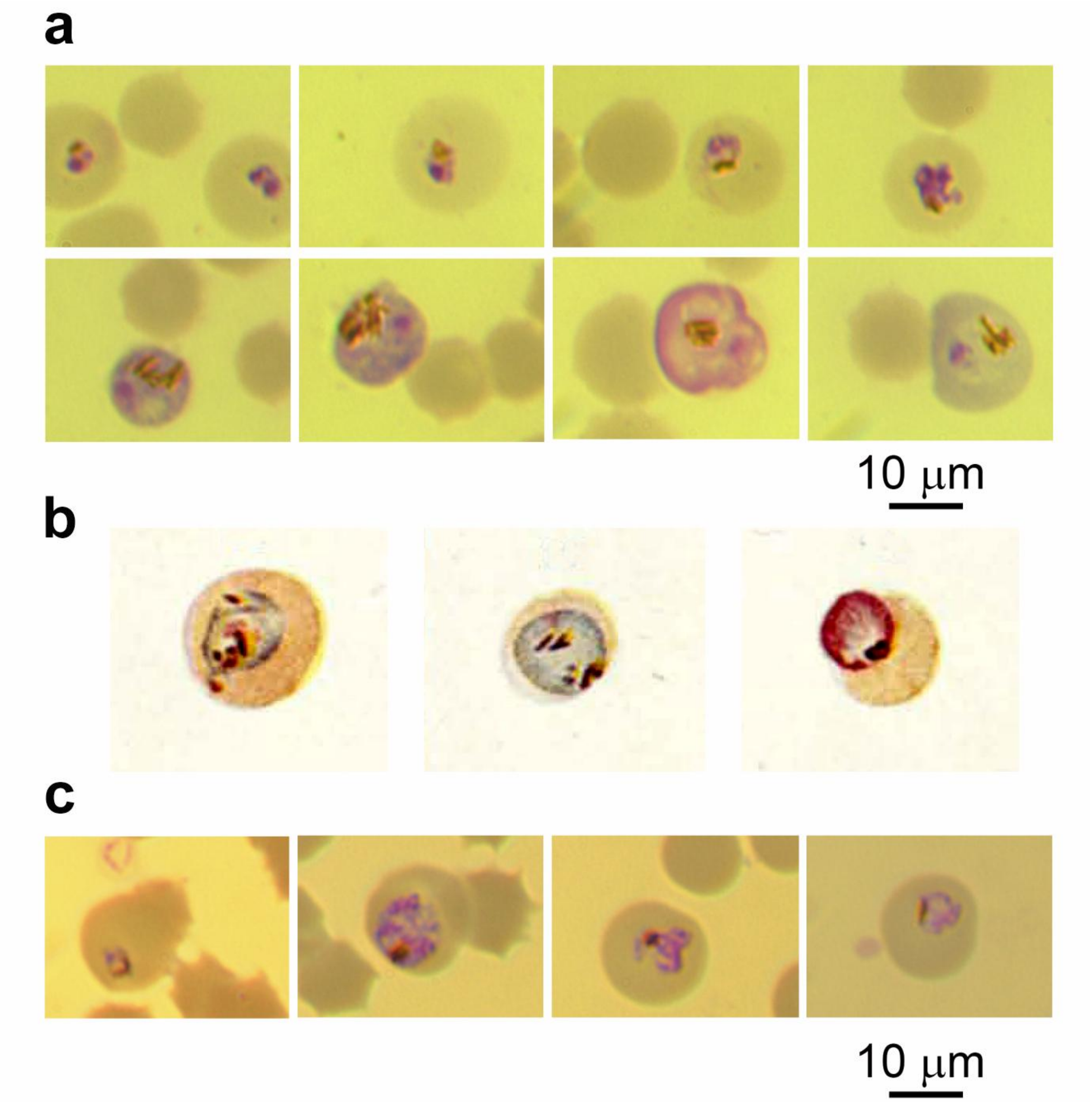

Supplementary Figure S1. Additional bright field and hand-drawn images of Giemsa reagent-stained parasites. Images are of infected erythrocytes within blood smears from (**a**), blood sample from a sick water buffalo in the Chachoengsao Province of Thailand in 2008; (**b**), select hand-drawn images reproduced from Sheather, 1919[1] showing bar-shaped hemazoin; and (**c**), blood sample from a water buffalo in the Mukdahan Province of Thailand in 2015. Type-specific PCR of DNA isolated from the blood of this buffalo indicated that the parasite is Type I.

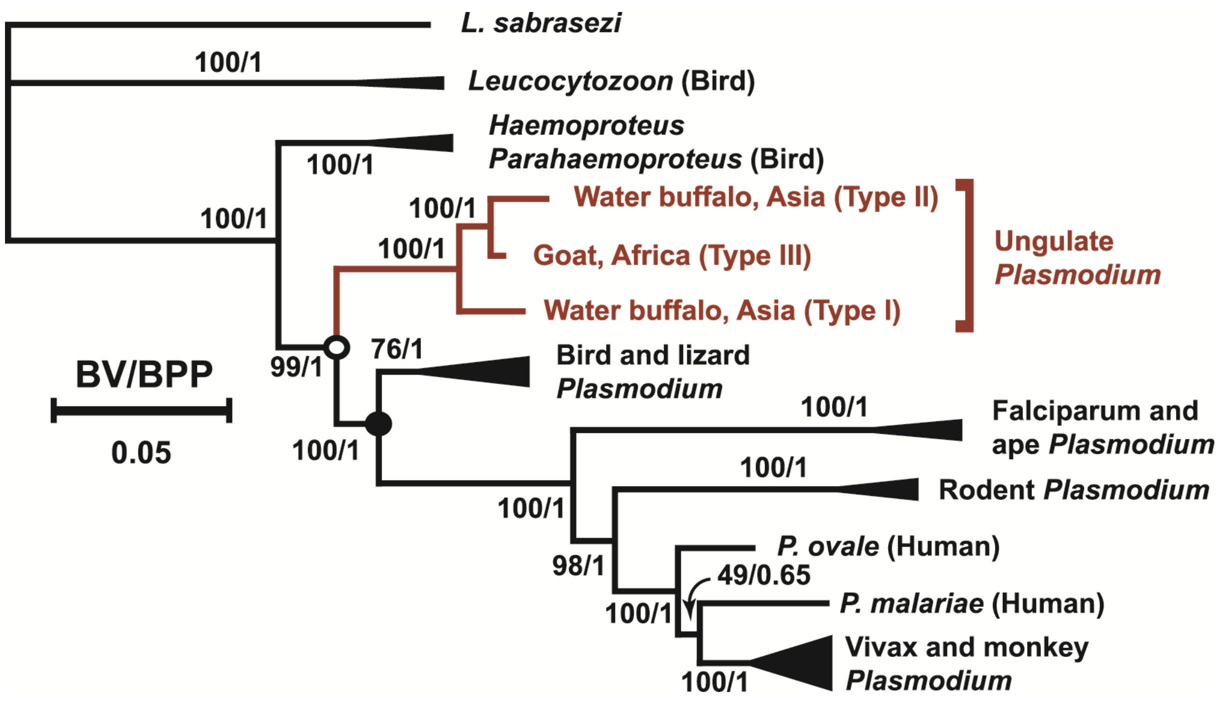

Supplementary Figure S2. Phylogenetic relationships of ungulate *Plasmodium* within the Haemosporida (codon model). Phylogenetic analysis was also conducted after partitioned into the non-coding and coding region, to which a nucleotide model (TVM+I+G) and a codon model were employed, respectively. For the codon model-based analysis, a model proposed by Goldman and Yang, 1994[2] was applied with the +I and +G options, as the frequency of each codon was also estimated from the data (+F option). We found that this partitioned model-based analysis showed smaller AIC scores compared to the concatenated model-based analysis employed to infer the tree shown in Fig. 2. The ungulate *Plasmodium* groups together and branches before diversification of malaria parasites that infect bird, lizard, rodent, monkey, apes and human; with high bootstrap and BPP values. The tree was inferred by the maximum likelihood (ML) method using 36 whole mitochondria genome sequences partitioned into non-coding and coding regions. The length for the substitutions/site (0.05) is indicated. Bootstrap values (BV) by ML with 1,000 replicates and Bayesian posterior probability (BPP) are indicated for each internal branch. Collapsed clades are indicated, such as "Vivax and monkey *Plasmodium*" clade, and their compositions are described in the Methods section.

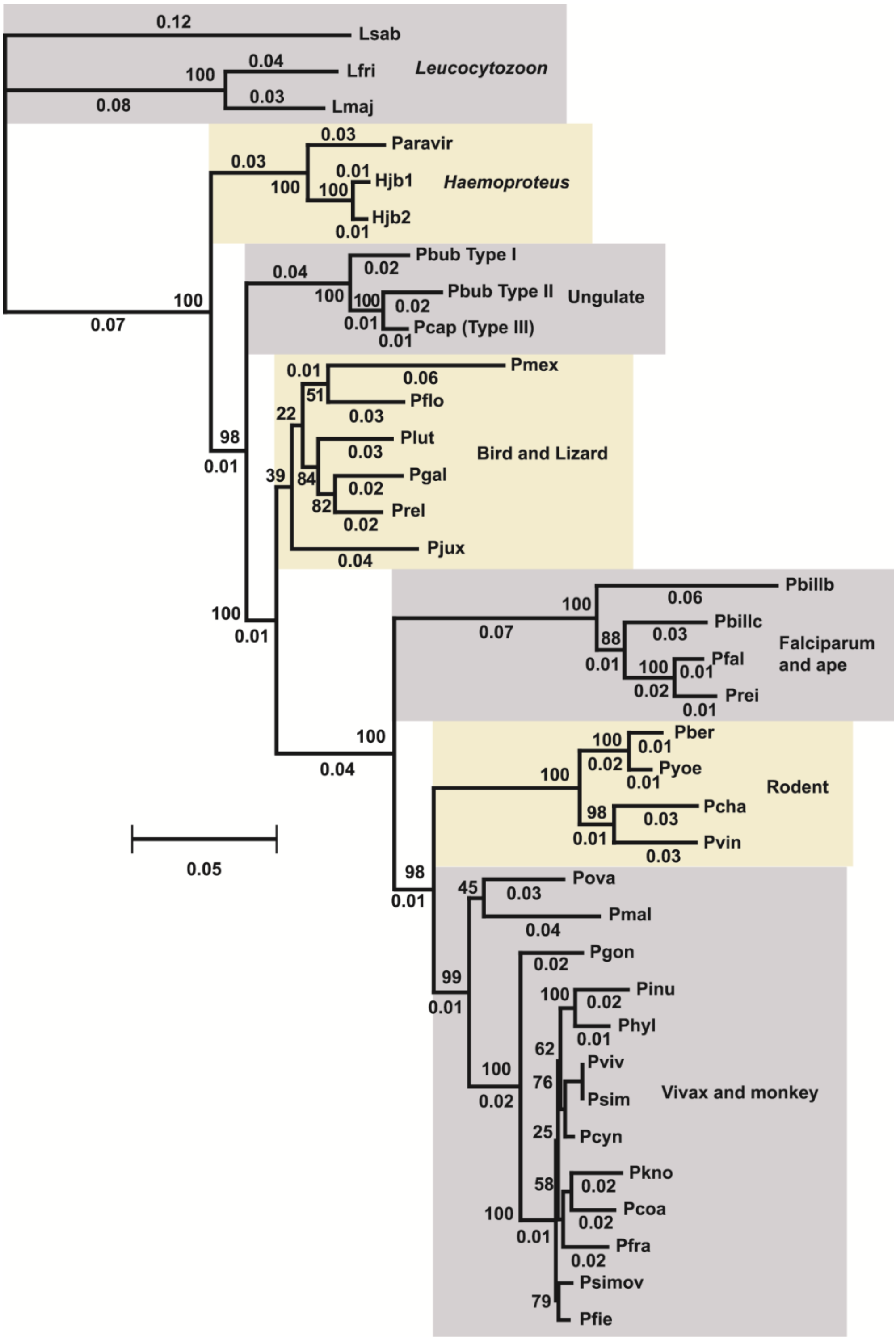

Supplementary Figure S3. Phylogenetic relationships of ungulate *Plasmodium* within the Haemosporida with an uncollapsed tree. To compare the branch lengths, the collapsed clades in Figure 2 are expanded and the branch lengths (substitutions/site) are indicated for each branch. Species abbreviations are given in Table S1. Bootstrap values by ML are indicated for each internal branch.

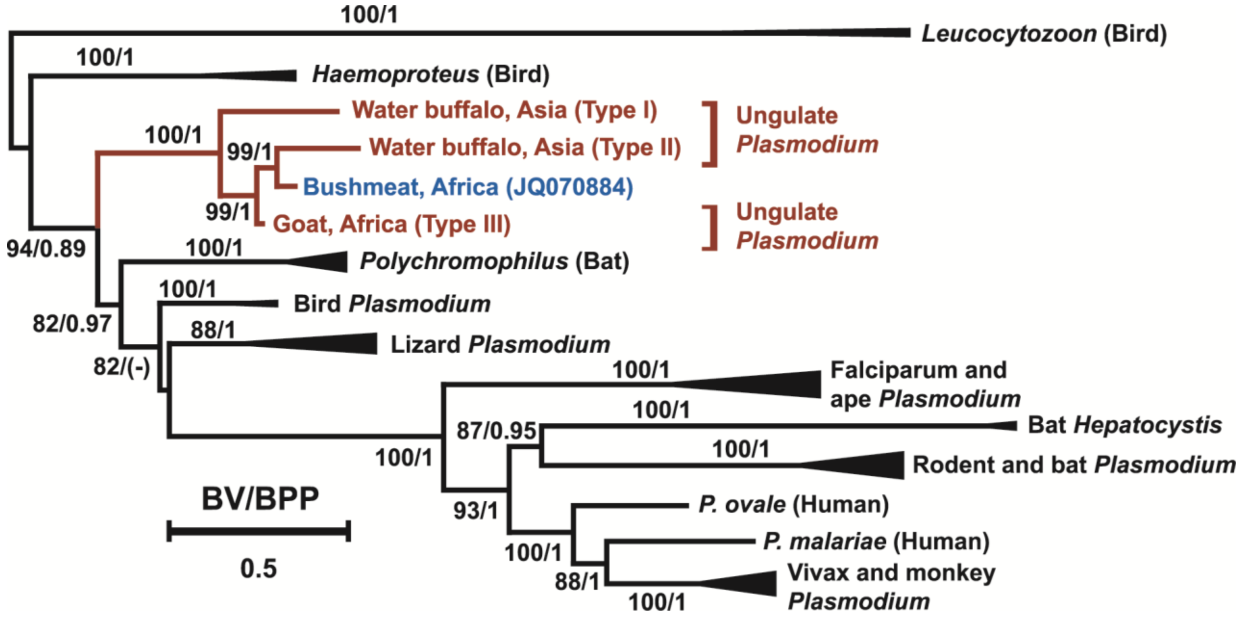

Supplementary Figure S4. Sequence obtained from a bushmeat in the previous report (JQ070884) is phylogenetically related to the ungulate *Plasmodium* sequences. The tree was inferred by the maximum likelihood (ML) method based on the codon (GY+I+G+F) model using concatenated codon sequences of *coxI*, *cytb*, and *clpc* genes derived from 52 *Plasmodium* and the *cytb* sequence obtained from the related parasites of a bushmeat (JQ070884). 0.5 indicates substitutions/site. Gaps existing in equal to or greater than 50% of the sequences were excluded from the analysis. Bootstrap values (BV) by ML with 1,000 replicates and Bayesian posterior probability (BPP) are indicated for each internal branch. Collapsed clades are indicated, such as "Vivax and monkey *Plasmodium*" clade, and their compositions are described in Table S1.

Supplementary Table S1 | Nucleotide and amino acid sequences used for the phylogenetic analyses[a]

Grouping, parasite and abbreviation	Host	Host species	*cox1*	*cytb*	*clpc*	mtDNA
Leucocytozoon						
Leucocytozoon fringillinarium (Lfri)	Bird	*Pipilo chlorurus*				FJ168564
Leucocytozoon majoris (Lmaj)	Bird	*Zonotrichia leucophrys ori.*				FJ168563
Leucocytozoon sabrasezi (Lsab)	Bird	*Gallus gallus*				AB299369
Leucocytozoon sp. (2109)	Bird	*Buteo jamaicensis*	EU254563	EU254518	EU254609	
Leucocytozoon sp. (157)	Bird	*Accipiter brevipes*	EU254564	EU254519	EU254610	
Leucocytozoon sp. (2208)	Bird	*Buteo lineatus*	EU254565	EU254520	EU254611	
Haemoproteus/Parahaemoproteus						
Haemoproteus sp. jb1.JA27 (Hjb1)	Bird	*Meliphaga lewinii*				AY733086
Haemoproteus sp. jb2.SEW5141 (Hjb2)	Bird	*Lichenostomus frenatus*				AY733087
Parahaemoproteus vireonis (Paravir)	Bird	*Vireo gilvus*				FJ168561
Haemoproteus belopolskyi	Bird	*Sylvia curruca*	EU254603	DQ451408	EU254657	
Haemoproteus coatneyi	Bird	*Dendroica coronata*	EU254595	EU254550	EU254648	
Haemoproteus turtur	Bird	*Streptopelia senegalensis*	EU254592	DQ451425	EU254644	
Bird *Plasmodium*						
Plasmodium gallinaceum (Pgal)	Bird	*Gallus gallus*	AB564275	AY099029	AB649424	AB250690
Plasmodium relictum (Prel)	Bird	*Hemignathus virens*	AY733090	AY733090	EU254633	AY733090
Plasmodium juxtinucleare (Pjux)	Bird	*Gallus gallus*				AB250415
Plasmodium lutzi (Plut)	Bird	*Turdus fuscater*				KC138226
Lizard *Plasmodium*						
Plasmodium floridense (Pflo)	Lizard	*Anolis oculatus*	EF079654	EF079654	EU254620	GQ355469
Plasmodium azurophilum	Lizard	*Anolis oculatus*	EU254575	EU254532	EU254622	
Plasmodium chiricahuae	Lizard	*Sceloporus jarrovi*	KF049536	AY099061	KF049558	
Plasmodium giganteum	Lizard	*Agama agama*	EU254577	AY099053	EU254624	
Plasmodium leucocytica	Lizard	*Anolis oculatus*	EU254576	EU254533	EU254623	
Plasmodium mexicanum (Pmex)	Lizard	*Sceloporus occidentalis*	EF079653	EF079653	EU254619	EF079653
Human *Plasmodium*						
Plasmodium falciparum (Pfal)	Primate	Human	M76611	DQ642845	DQ642846	NC_002375
Plasmodium vivax (Pviv)	Primate	Human	AY598140	AY598140	AF348344	AY598140
Plasmodium malariae (Pmal)	Primate	Human	AB489193	AF069624	AF348342	AB354570
Plasmodium ovale (Pova)	Primate	Human	JF894415	AF069625	AY634623	AB354571
Monkey *Plasmodium*						
Plasmodium coatneyi (Pcoa)	Primate	Old world monkeys	AB354575	EU400407	AB471872	AB354575
Plasmodium cynomolgi (Pcyn)	Primate	Old world monkeys	AB444126	AF069616	AB471873	AB434919
Plasmodium fieldi (Pfie)	Primate	Old world monkeys	AB354574	AB354574	AB471874	AB354574
Plasmodium gonderi (Pgon)	Primate	Old world monkeys	AB434918	AF069622	AB471877	AB434918
Plasmodium inui (Pinu)	Primate	Old world monkeys	AB354572	AF069617	AB471879	HM032052
Plasmodium knowlesi (Pkno)	Primate	Old world monkeys	AY598141	AF069621	AF348341	AY722797
Plasmodium fragile (Pfra)	Primate	Old world monkeys				AY722799

Supplementary Table S1 (continued) | Nucleotide and amino acid sequences used for the phylogenetic analyses[a]

**Grouping, parasite and abbreviation**	**Host**	**Host species**	***cox1***	***cytb***	***clpc***	**mtDNA**
Plasmodium simiovale (Psimov)	Primate	Old world monkeys				AB434920
Plasmodium simium (Psim)	Primate	Old world monkeys				AY722798
Plasmodium hylobati (Phyl)	Primate	*Hylobates moloch*	AB354573	AB354573	AB471878	AB354573
Ape *Plasmodium*						
Plasmodium reichenowi (Prei)	Primate	*Pan troglodytes*	AJ251941	AJ251941	EU560464	AJ251941
Plasmodium billcollinsi (Pbillc)	Primate	*Pan troglodytes*				GQ355479
Plasmodium billbrayi (Pbillb)	Primate	*Pan troglodytes*				GQ355469
Plasmodium gaboni	Primate	*Pan troglodytes*	FJ895307	FJ895307	HQ842630	
Plasmodium sp. C2	Primte	*Pan troglodytes*	HM235400	HM235404	HM235145	
Plasmodium sp. C3	Primate	*Pan troglodytes*	HM235360	HM235360	HM235151	
Plasmodium sp. G1	Primate	*Gorilla gorilla*	HM235308	HM235288	HM235163	
Plasmodium sp. G2	Primate	*Gorilla gorilla*	HM235383	HM234984	HM235148	
Plasmodium sp. G3	Primate	*Gorilla gorilla*	HM235290	HM234998	HM235154	
Rodent *Plasmodium*						
Plasmodium berghei (ANKA) (Pber)	Rodent	*Grammomys surdaster*	DQ414589	DQ414645	DQ417612	AB558173
Plasmodium chabaudi (Pcha)	Rodent	*Thamnomys rutilans*	DQ414594	AY099050	DQ417617	AF014116
Plasmodium vinckei (Pvin)	Rodent	*Grammomys surdaster*	DQ414596	DQ414651	DQ417619	AB599931
Plasmodium yoelii (17X) (Pyoe)	Rodent	*Thamnomys rutilans*	DQ414605	AY099051	DQ417628	M29000
Plasmodium atheruri	Rodent	*Atherurus africanus*	DQ414588	AY099054	DQ417611	
Bat *Plasmodium*						
Plasmodium cyclopsi L1_1	Bat	*Hipposideros cyclops*	KF159788	KF159710	KF159635	
Plasmodium cyclopsi L4_1	Bat	*Hipposideros cyclops*	KF159789	KF159674	KF159630	
Plasmodium cyclopsi L4_2	Bat	*Hipposideros cyclops*	KF159791	KF159716	KF159637	
Plasmodium voltaicum G1_1	Bat	*Myonycteris angolensis*	KF159792	KF159671	KF159648	
Bat *Hepatocystis*						
Hepatocystis sp.MB6	Bat	*Nanonycteris veldkampii*	EU254570	EU254527	EU254617	
Hepatocystis sp.MB3	Bat	*Nanonycteris veldkampii*	EU254571	EU254528	EU254618	
Hepatocystis sp.	Primate	*Cercopithecus nictitans*		JQ070884		
Polychromophilus						
Polychromophilus melanipherus Type 3	Bat	*Miniopterus schreibersii*	JN990714	JN990708	JN990720	
Polychromophilus melanipherus Type 4	Bat	*Miniopterus schreibersii*	JN990715	JN990709	JN990721	
Polychromophilus melanipherus Type 5	Bat	*Miniopterus schreibersii*	JN990716	JN990710	JN990722	
Polychromophilus. sp. G3_2	Bat	*Miniopterus villiersi*	KF159795	KF159699	KF159616	
Polychromophilus. sp. G3_3	Bat	*Miniopterus villiersi*	KF159796	KF159681	KF159642	
Polychromophilus sp. G3_1	Bat	*Pipistrellus aff. grandidieri*	KF159797	KF159714	KF159639	

[a] Mitochondrial DNA and *clpc* DNA nucleotide sequences determined from ungulate *Plasmodium* in this study were deposited in DDBJ/EMBL/GenBank with the accession numbers LC090213 - LC090217.
